# The impact of hormonal fluctuations and the efficacy of targeted hormonal interventions in improving macular hole outcomes

**DOI:** 10.3389/fmed.2025.1652028

**Published:** 2025-11-10

**Authors:** Lisha Ni, Liwei Ma, Minghua Fan, Lina Zhang

**Affiliations:** Department of Ophthalmology, Lishui Hospital of Wenzhou Medical University, The First Affiliated Hospital of Lishui University, Lishui People's Hospital, Lishui, Zhejiang, China

**Keywords:** macular hole, estrogen replacement therapy, testosterone supplementation, hormonal fluctuations, retinal thickness

## Abstract

**Background:**

Macular hole formation is influenced by vitreoretinal dynamics, with hormonal status playing a critical role in retinal stability. Reduced estrogen and testosterone levels in postmenopausal women and men with hypogonadism have been linked to increased macular hole severity. This study investigates the impact of hormonal fluctuations and the efficacy of targeted hormonal interventions in improving macular hole outcomes.

**Methods:**

A total of 118 participants were divided into four groups: premenopausal women (*n* = 30), postmenopausal women (*n* = 35), men with normal testosterone levels (*n* = 28), and men with hypogonadism (*n* = 25). Hormonal profiles were measured using LC–MS/MS, and macular hole severity was assessed with SD-OCT. Hormonal interventions included estrogen replacement therapy (ERT) and testosterone supplementation. Statistical analysis involved ANOVA, chi-square tests, and regression models.

**Results:**

Participants with low hormone levels exhibited larger macular holes and higher progression rates. Postmenopausal women without ERT and men with hypogonadism had progression rates of 55 and 60%, respectively, while those receiving hormonal interventions showed reduced rates of 30 and 35%. Hormonal interventions significantly improved macular hole closure rates (70% in ERT and 65% in testosterone groups) and visual acuity gains (2.8 lines and 2.5 lines, respectively). Subgroup analysis indicated better outcomes in early postmenopausal women and younger men, while diabetic participants showed poorer results.

**Conclusion:**

Hormonal fluctuations significantly impact macular hole progression, and hormonal interventions can enhance closure rates and visual outcomes. Early hormonal therapy initiation is associated with better results, highlighting the potential for personalized treatment strategies.

## 1 Introduction

A macular hole is a full-thickness defect in the central retina, specifically in the macula, which is responsible for sharp, central vision. A full-thickness macular hole (FTMH) significantly impairs visual acuity and often leads to central vision loss, affecting activities such as reading and recognizing faces. Surgical intervention, particularly pars plana vitrectomy, remains the gold standard treatment to improve visual function and promote anatomical closure. Patients often present with symptoms such as blurring of central vision, distortion (metamorphopsia), and central scotomas, which can significantly impact daily activities such as reading and driving. While spontaneous closure of macular holes can occur, especially in small stage 1 lesions, it is relatively rare and unpredictable. Therefore, most cases, particularly FTMHs, require active treatment. The most common risk factors include advancing age, female gender, and ocular trauma. Macular holes are classified into stages, from early foveal detachment (stage 1) to FTMHs (stages 2 to 4), which may lead to irreversible visual loss if left untreated (1).

Surgical intervention, primarily pars plana vitrectomy, has proven effective in closing macular holes and improving visual outcomes. However, not all patients achieve full visual recovery, highlighting the need to explore additional biological factors influencing macular hole formation and progression. Beyond mechanical vitreoretinal traction, biochemical changes in the vitreous and retina may also contribute to the disease, suggesting a multifactorial etiology (2).

Epidemiological studies consistently show a higher prevalence of macular holes in women, particularly after menopause. This gender disparity raises the question of whether sex hormones, especially estrogen and progesterone, play a role in retinal health and disease. Estrogen is known to influence various tissues, including ocular structures, through its effects on collagen metabolism, vascular tone, and inflammation. The postmenopausal decline in estrogen levels has been implicated in other ocular conditions, such as dry eye and age-related macular degeneration (AMD), making it a potential factor in macular hole formation as well.

Epidemiological studies consistently demonstrate a higher prevalence of macular holes among women, particularly after menopause, suggesting a potential hormonal influence. Sex hormones such as estrogen and progesterone are known to modulate retinal and vitreous physiology through pathways involving collagen remodeling, vascular regulation, and anti-inflammatory signaling. The postmenopausal decline in estrogen has been linked to ocular degenerative changes, including altered vitreoretinal adhesion and reduced retinal resilience, as reported in recent studies. These observations collectively indicate that hormonal fluctuations may play a mechanistic role in macular hole pathogenesis (3, 4).

Furthermore, studies have shown that hormonal fluctuations may influence vitreous collagen remodeling and its adhesion to the retinal surface. Estrogen and progesterone receptors have been identified in retinal pigment epithelial and Müller glial cells, suggesting a direct hormonal role in maintaining retinal structural integrity. Despite these insights, the mechanisms linking systemic hormonal decline to macular hole formation remain insufficiently characterized, highlighting the need for further research. Understanding these pathways could inform preventive strategies and adjunctive therapies for high-risk populations, particularly postmenopausal women (5).

.Several studies have previously explored the relationship between plasma sex hormones such as estrogen, progesterone, and testosterone and macular hole formation, often highlighting a higher prevalence of macular holes in postmenopausal women. These investigations have largely focused on establishing correlations between hormonal changes and the increased risk of developing macular holes. While these findings have provided valuable insights, they remain primarily observational, offering limited understanding of the underlying biological mechanisms. Simple correlation studies, though important, do not adequately explain how plasma sex hormones influence the structural integrity of the retina and vitreous or the precise pathways through which hormonal fluctuations may contribute to macular hole development (6).

Moreover, existing research has seldom examined the potential for hormone-based therapeutic interventions to prevent macular holes or improve surgical outcomes following vitrectomy. The lack of mechanistic insights into hormonal regulation of vitreoretinal dynamics represents a critical gap in knowledge. Understanding how sex hormones interact with retinal tissues and the vitreous matrix at the molecular level is essential for developing novel preventive strategies and targeted treatments (7).

Given these limitations, there is a pressing need for innovative research that goes beyond simple correlation. Investigating the precise mechanisms by which plasma sex hormones influence macular hole pathogenesis could provide a deeper understanding of disease progression. Additionally, evaluating the efficacy of hormone-based therapies, such as estrogen replacement or testosterone supplementation, as adjunctive treatments represent a promising area of clinical exploration. Bridging these gaps in knowledge could pave the way for more personalized and effective approaches to macular hole management, especially in high-risk populations (4).

The primary aim of this study was to evaluate the impact of plasma sex hormone levels on macular hole severity and progression in different hormonal groups, including premenopausal women, postmenopausal women, men with normal testosterone levels, and men with hypogonadism. Additionally, the study sought to investigate the relationship between hormonal fluctuations and macular hole progression, with a specific focus on participants experiencing high hormonal variability, such as postmenopausal women without estrogen replacement therapy (ERT) and men with hypogonadism. Another key objective was to assess the efficacy of targeted hormonal interventions, including ERT in postmenopausal women and testosterone supplementation in men with hypogonadism, in reducing macular hole progression and improving structural and functional retinal outcomes. The study further aimed to compare macular hole closure rates, changes in minimum linear diameter (MLD), retinal thickness, and visual acuity improvements between intervention and control groups. Subgroup analyses were performed to explore how various factors, such as menopausal duration, age, and comorbidities, particularly diabetes, influenced the effectiveness of hormonal interventions. The ultimate goal was to identify critical factors affecting treatment outcomes and develop personalized therapeutic strategies for patients with macular holes, emphasizing the potential role of early hormonal therapy initiation in improving clinical results.

## 2 Materials and methods

### 2.1 Study design and participant recruitment

This prospective observational cohort study was conducted over a 12-month period (January 2022 to January 2023) to investigate the association between plasma sex hormone levels and macular hole severity. A total of 118 participants were recruited and categorized into four groups based on hormonal status: premenopausal women (*n* = 30), postmenopausal women (*n* = 35), men with normal testosterone levels (*n* = 28), and men with hypogonadism (*n* = 25). Recruitment was carried out at the Department of Ophthalmology, Lishui People’s Hospital, Zhejiang Province, China. Eligible patients were identified through the hospital’s outpatient retina clinic and surgical referral lists. Potential participants were screened based on clinical presentation and spectral-domain optical coherence tomography (SD-OCT) findings, and those meeting inclusion criteria were invited to participate. Written informed consent was obtained from all participants prior to enrollment. The study protocol was reviewed and approved by the Institutional Review Board (IRB) of the participating institutions and conducted in accordance with the Declaration of Helsinki.

All surgical procedures were centralized at Lishui People’s Hospital to ensure consistency in technique and quality control. All participants underwent standard 25-gage pars plana vitrectomy (PPV) with internal limiting membrane (ILM) peeling performed by the same two experienced vitreoretinal surgeons. The surgical procedure included induction of posterior vitreous detachment when necessary, ILM peeling with indocyanine green (ICG) dye, and 20% sulfur hexafluoride (SF₆) gas tamponade. Postoperatively, all patients were instructed to maintain a face-down position for 5 days. Centralizing surgeries and standardizing personnel minimized inter-surgeon variability and ensured procedural uniformity across cases.

Diabetes mellitus was defined according to the American Diabetes Association (ADA) 2023 criteria as any of the following: (1) a documented diagnosis in the participant’s medical record; (2) current use of antidiabetic medication (oral hypoglycemics or insulin); or (3) a hemoglobin A1c (HbA1c) level ≥6.5% measured within the preceding 3 months. Diabetes status was verified using electronic medical records and laboratory data rather than self-reported history. The duration of visual symptoms prior to surgery was recorded based on clinical history and later considered in subgroup analyses to evaluate its potential role as a confounder of surgical outcomes.

Participants were eligible for inclusion if they were aged 30–65 years and had a confirmed diagnosis of macular hole based on SD-OCT imaging. Exclusion criteria included a history of ocular surgery, presence of other retinal diseases that could influence outcomes, or use of hormone therapy or androgen suppression within the previous 6 months. Individuals with systemic diseases known to affect hormone levels (e.g., thyroid disorders) were excluded, except for those with hypogonadism in the designated group.

Exclusion criteria included a history of ocular trauma, retinal detachment, previous vitreoretinal surgery, secondary macular holes, high myopia (axial length ≥ 26.5 mm or refractive error ≤ − 6.0 D), and the use of hormone therapy or androgen suppression therapy within the past 6 months.

Fasting blood samples for hormone analysis were collected between 8:00 and 10:00 a.m. to minimize diurnal variation. In premenopausal women, sampling was standardized to the early follicular phase (days 3–7) of the menstrual cycle to reduce inter-cycle variability, and urine pregnancy testing was performed at enrollment to exclude pregnancy-related hormonal fluctuations. Plasma estrogen, progesterone, and testosterone concentrations were quantified using liquid chromatography–tandem mass spectrometry (LC–MS/MS), a highly sensitive and specific method for low-concentration hormone measurement. All assays were performed at a certified clinical laboratory following standardized procedures to ensure analytical accuracy and reproducibility.

Baseline ocular imaging was conducted using SD-OCT to evaluate macular hole characteristics. The MLD was measured in micrometers (μm) by a single trained technician using the same SD-OCT device for all participants to minimize inter-device variability. Two independent masked graders assessed all images, and any discrepancies were resolved by consensus with a third reviewer.

### 2.2 Study design

This longitudinal observational study evaluated the impact of hormonal fluctuations on macular hole progression and retinal structural changes over a 12-month follow-up. Participants underwent serial hormone measurements and ocular assessments at baseline, 6 months, and 12 months. To capture variability in hormonal status, participants were stratified into four groups: postmenopausal women without estrogen replacement therapy (ERT), postmenopausal women receiving ERT, men with hypogonadism, and men with normal testosterone levels. This stratification enabled accurate evaluation of the influence of hormonal milieu and treatment status on disease progression.

Hormonal variability was quantified using a Hormone Variability Index (HVI), a modified metric adapted from variability analyses commonly applied in endocrine research (6, 7). The HVI represents intra-individual percentage variability in hormone levels across the follow-up period and was calculated as:


$$\mathrm{HVI}=\frac{\text{Standard Deviation of Hormone Levels Across Visits}}{\text{Mean Hormone Level Across Visits}}\times 100$$

This coefficient-of-variation based approach reflects relative hormonal instability during follow-up, where higher HVI values denote greater fluctuation. The HVI was calculated separately for estrogen, progesterone, and testosterone, and averaged to yield a composite score. Plasma hormone concentrations were determined at each visit using liquid chromatography–tandem mass spectrometry (LC–MS/MS), following standardized laboratory protocols to ensure precision and reproducibility.

Macular hole progression was monitored using SD-OCT. Structural parameters, including minimum MLD and CRT (both measured in micrometers), were evaluated at each visit by a single trained technician using the same device to maintain consistency. All images were independently reviewed by two masked graders, and any discrepancies were resolved by consensus.

Participants receiving hormonal therapy followed standardized regimens: oral estradiol (2 mg/day) for postmenopausal women and intramuscular testosterone (100 mg every 2 weeks) for men with hypogonadism. Adherence was monitored at follow-up visits through clinical interviews and treatment records to ensure protocol compliance.

### 2.3 Study design: hormonal interventions

This component of the study was a randomized controlled trial (RCT) conducted to evaluate the efficacy of hormonal interventions in improving macular hole outcomes. Participants were randomly assigned to intervention or control groups using a computer-generated allocation sequence to ensure concealment. Postmenopausal women were allocated to either an estrogen replacement therapy (ERT) group or a placebo group, while men with hypogonadism were assigned to either a testosterone replacement therapy (TRT) group or a placebo control group.

Participants in the ERT arm received oral estradiol (2 mg/day), and those in the TRT arm received intramuscular testosterone injections (100 mg every 2 weeks). Both interventions were administered for 12 months. Placebo controls received identical capsules or inert saline injections to maintain blinding and procedural consistency across treatment arms.

Treatment adherence was monitored through monthly follow-up visits and self-reported compliance logs, and any deviations from the assigned regimen were documented and considered in subsequent analyses.

Macular hole outcomes were evaluated at baseline, 6 months, and 12 months using standardized procedures described previously (Section 2.1). The primary endpoints included macular hole closure rate, minimum MLD, CRT, and visual acuity (VA) improvement measured with a Snellen chart. All imaging and measurements followed uniform SD-OCT protocols to ensure methodological consistency.

### 2.4 Subgroup stratification

To examine variability in macular hole outcomes across different populations, subgroup analyses were conducted among participants receiving hormonal interventions. Stratification was performed within each treatment group based on menopausal duration, age, and comorbidities. Among women, only postmenopausal participants were eligible for estrogen replacement therapy (ERT) and were further stratified by menopausal duration (<5 years vs. ≥5 years). Premenopausal women, who did not receive hormonal intervention, served as a reference group for comparative analysis. Among men, only those with hypogonadism were eligible for testosterone supplementation, and were stratified according to age (<50 years vs. ≥50 years) to capture potential age-related differences in hormonal response. In both sexes, comorbidities such as diabetes mellitus were also considered, with participants classified as diabetic or non-diabetic based on verified medical and laboratory records.

All participants in the hormonal intervention groups received estrogen replacement therapy (ERT) (for postmenopausal women) or testosterone supplementation (for hypogonadal men) according to standardized clinical regimens and baseline hormonal profiles.

Hormonal therapy was initiated 2 weeks prior to vitrectomy and maintained for 6 months postoperatively to ensure stable systemic hormone levels throughout the perioperative and recovery periods. Follow-up visits were conducted at baseline, 3 months, 6 months, and 12 months, during which serum hormone assays, ocular imaging, and visual acuity assessments were performed to monitor adherence, hormonal stability, and treatment outcomes (SD-OCT) imaging, and visual acuity testing were performed according to uniform study protocols.

Macular hole outcomes were assessed using high-resolution SD-OCT following the same imaging and grading procedures outlined in Section 2.1. The primary endpoints included macular hole closure rate, defined as complete anatomical closure on SD-OCT; minimum MLD and CRT, both measured in micrometers (μm) to evaluate structural changes; and VA improvement, determined using a Snellen chart and expressed as the number of lines gained from baseline.

This stratified approach allowed for a comprehensive assessment of how menopausal duration, age, and diabetic status influenced the efficacy of hormonal interventions on macular hole repair and visual recovery.

### 2.5 Statistical analysis

All statistical analyses were performed using SPSS version 25.0 (IBM Corp., Armonk, NY, United States) and GraphPad Prism version 9.0 (GraphPad Software, San Diego, CA, USA). Descriptive statistics were used to summarize participant demographics, hormonal profiles, and ocular parameters. Continuous variables were expressed as mean ± standard deviation (SD), and categorical variables were presented as proportions.

Differences among the primary groups—premenopausal women, postmenopausal women, men with normal testosterone, and men with hypogonadism—were assessed using one-way analysis of variance (ANOVA). When significant main effects were observed, Tukey’s honestly significant difference (HSD) post-hoc tests were applied to determine pairwise intergroup differences.

Repeated-measures ANOVA was employed to evaluate longitudinal changes in hormone levels, MLD, and CRT across baseline, 6-month, and 12-month follow-up visits. Hormonal variability over time was quantified using the Hormone Variability Index (HVI), and its association with macular hole progression was analyzed through linear regression models.

Comparisons between hormonal intervention and control groups were conducted to assess differences in macular hole closure rates, retinal morphology, and visual acuity (VA) outcomes. Chi-square tests were used for categorical comparisons such as closure rate, while paired t-tests examined within-group changes in MLD, CRT, and VA across time points.

Subgroup analyses were performed to investigate outcome variability according to menopausal duration, age, and comorbidities. Two-way ANOVA with Bonferroni correction for multiple comparisons was applied to evaluate interaction effects between subgroup characteristics and hormonal interventions. In addition, Pearson’s correlation analysis explored associations between hormone levels and ocular outcomes, and multivariate regression models were constructed to identify predictors of macular hole closure based on baseline hormone levels and variability indices.

Statistical significance was set at *p* < 0.05, and 95% confidence intervals (CI) were reported for all regression estimates. Graphical outputs, including bar charts and line graphs, were generated to visualize key comparisons and trends. This comprehensive analytical approach ensured robust interpretation of the data and reliable identification of factors influencing macular hole progression and visual recovery.

## 3 Result

### 3.1 Baseline demographics and hormonal profiles

A total of 118 participants were included in the study, divided into four groups based on hormonal status: premenopausal women (*n* = 30), postmenopausal women (*n* = 35), men with normal testosterone levels (*n* = 28), and men with hypogonadism (*n* = 25).

The mean age of participants varied across the groups: premenopausal women (35 ± 5 years), postmenopausal women (62 ± 6 years), men with normal testosterone levels (45 ± 8 years), and men with hypogonadism (55 ± 7 years). Baseline macular hole MLD also differed significantly across groups (*p* < 0.05): premenopausal women, 180 ± 35 μm; postmenopausal women, 270 ± 40 μm; men with normal testosterone, 210 ± 32 μm; men with hypogonadism, 300 ± 45 μm. Hormone levels and structural outcomes are summarized as mean ± SD. Regression analysis showed a positive association between hormone fluctuation index (HVI) and macular hole progression (*β* = 0.42, 95% CI 0.21–0.63, *p* = 0.001). Male participants with normal testosterone levels had a mean age of 45 ± 8 years, while hypogonadal men had a mean age of 55 ± 7 years, as shown in Table 1.

**Table 1:** Baseline demographics and hormonal profiles of participants.

Group	Participants (*n*)	Age (years, Mean ± SD)	Estrogen (pg/mL, Mean ± SD)	Progesterone (ng/mL, Mean ± SD)	Testosterone (ng/dL, Mean ± SD)	Macular Hole Severity MLD (μm, Mean ± SD)
Premenopausal women	30	35 ± 5	120 ± 25	10 ± 2.1	30 ± 6	180 ± 35
Postmenopausal women	35	62 ± 6	20 ± 5	1.5 ± 0.4	20 ± 5	270 ± 40
Men with normal testosterone	28	45 ± 8	25 ± 6	0.8 ± 0.2	550 ± 90	210 ± 32
Men with hypogonadism	25	55 ± 7	15 ± 4	0.5 ± 0.1	180 ± 40	300 ± 45
*p*-value*	—	<0.001	<0.001	<0.001	<0.001	<0.001

*p-values derived from one-way ANOVA comparing all four groups. MLD, Minimum linear diameter; SD, Standard Deviation.

**Figure 1:**
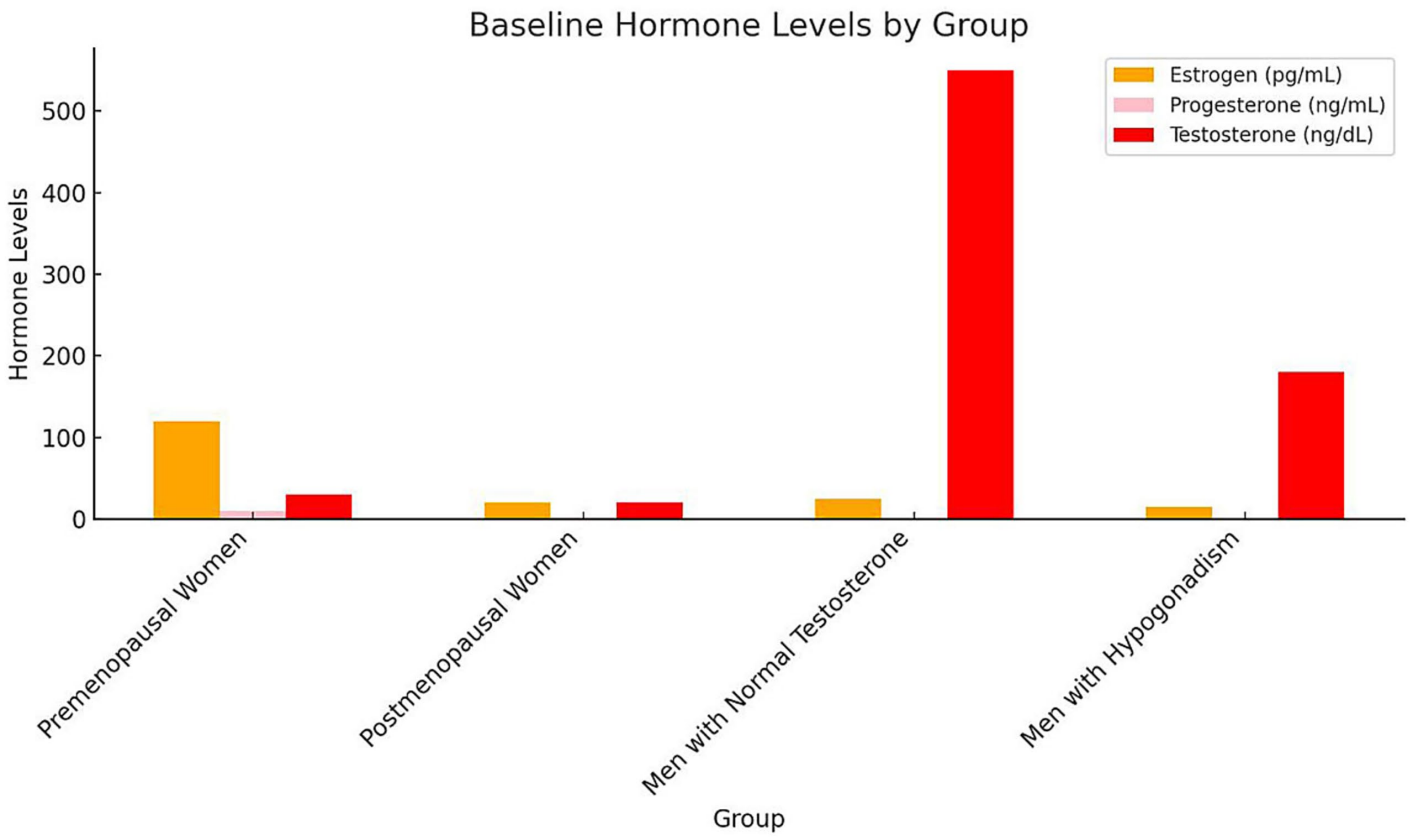
Baseline hormone levels by group bars represent mean ± SD concentrations of estrogen (pg/mL), progesterone (ng/mL), and testosterone (ng/dL) across the four study groups. Error bars indicate SD. Statistical differences among groups were evaluated using one-way ANOVA followed by Tukey’s post-hoc test (*p* < 0.05). Different letters above bars (a, b, c) denote significant inter-group differences (*p* < 0.05). Premenopausal women exhibited the highest estrogen and progesterone levels, whereas postmenopausal women and hypogonadal men showed significantly lower concentrations. Testosterone was highest in men with normal testosterone levels. SD, Standard deviation; ANOVA, Analysis of variance.

**Figure 2:**
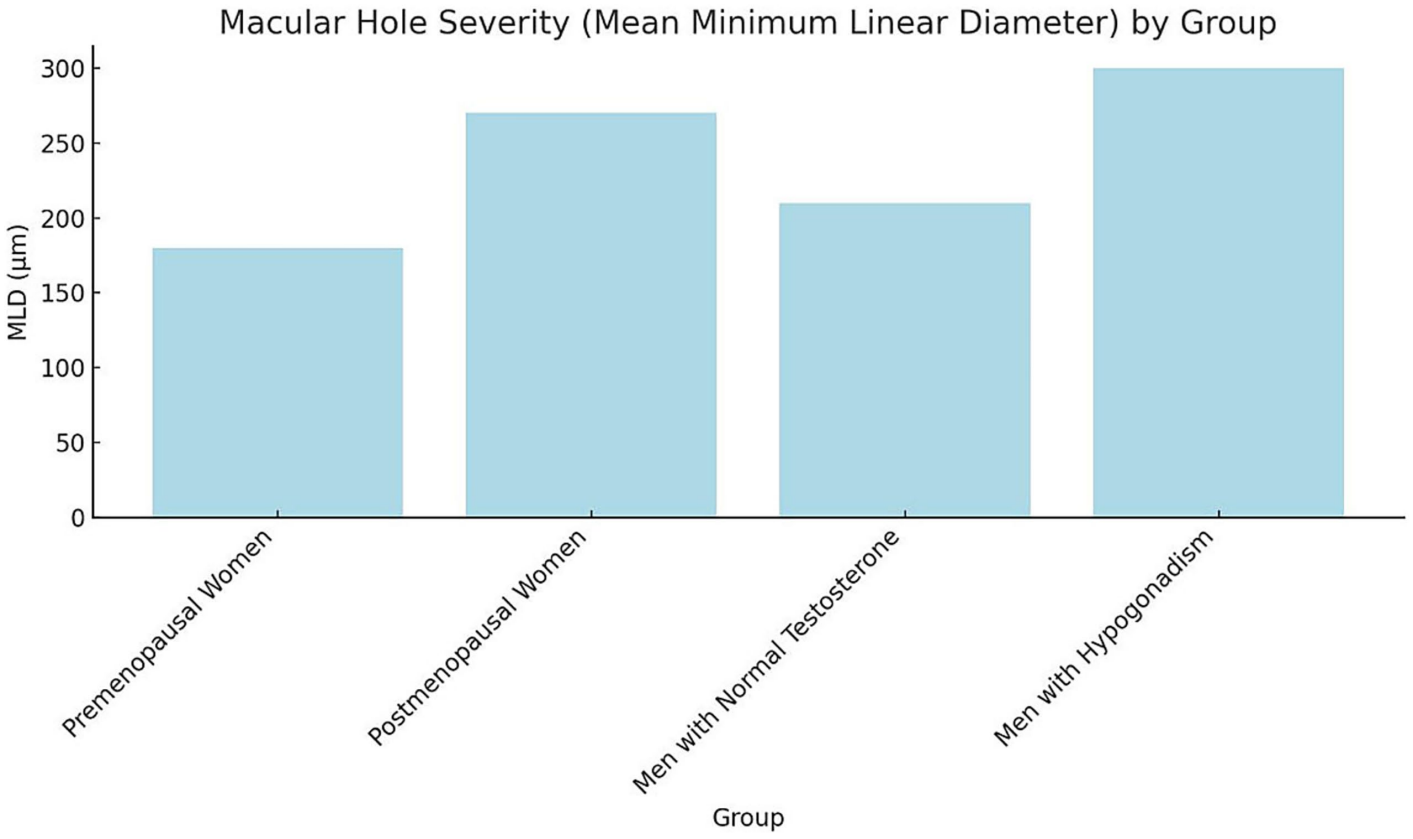
Macular hole severity (mean, MLD) by group bars represent mean ± SD of macular hole MLD (μm) across study groups. Error bars indicate SD. Statistical analysis was performed using one-way ANOVA followed by Tukey’s post-hoc test (*p* < 0.05). Groups sharing different letters (a, b, c) differ significantly (*p* < 0.05). SD, Standard deviation; MLD, Minimum linear diameter; ANOVA, Analysis of variance.

Baseline plasma hormone levels demonstrated notable differences among the groups. Premenopausal women exhibited the highest mean plasma estrogen levels (120 pg./mL), while postmenopausal women had significantly lower levels (20 pg./mL). Similarly, premenopausal women presented higher progesterone levels (10 ng/mL) compared to postmenopausal women (1.5 ng/mL). Among male participants, those with normal testosterone levels showed a mean plasma testosterone concentration of 550 ng/dL, substantially higher than that of hypogonadal men (180 ng/dL). As expected, estrogen and progesterone levels in male groups were comparably low, consistent with physiological norms for men (Figure 1). Statistical analysis confirmed these group differences were significant (*p* < 0.001, one-way ANOVA with Tukey’s post-hoc test). Hormone concentrations are expressed as mean ± SD: estrogen (premenopausal women 120 ± 25 pg./mL; postmenopausal women 20 ± 5 pg./mL), progesterone (10 ± 2.1 ng/mL vs. 1.5 ± 0.4 ng/mL), and testosterone (men with normal levels 550 ± 90 ng/dL vs. hypogonadal men 180 ± 40 ng/dL). These findings align with expected physiological patterns across sex and menopausal status. The severity of macular holes, assessed by mean MLD, varied significantly across hormonal groups (*p* < 0.001, one-way ANOVA; Figure 2). Premenopausal women exhibited smaller macular holes (180 ± 35 μm) compared to postmenopausal women (270 ± 40 μm, *p* < 0.01). Similarly, men with normal testosterone levels had lower MLDs (210 ± 32 μm) than hypogonadal men (300 ± 45 μm, *p* < 0.01). These results indicate that hormonal deficiency is associated with increased macular hole severity, suggesting that reduced estrogen or testosterone may contribute to structural retinal weakening and larger defects.

### 3.2 Impact of hormonal fluctuations on macular hole development

Analysis of the data revealed a significant association between hormonal fluctuations and macular hole progression. Participants experiencing high hormone fluctuations, such as postmenopausal women without estrogen replacement therapy (ERT) and men with hypogonadism, exhibited higher rates of macular hole progression and greater changes in retinal structure compared to participants with low hormone fluctuations (Table 2).

**Table 2:** Impact of hormonal variability on macular hole development.

Group	Participants (*n*)	Hormone Variability Index (HVI, Mean ± SD)	Macular Hole Progression Rate (%)	Change in MLD (μm, Mean ± SD)	Change in Retinal Thickness (μm, Mean ± SD)	*p*-value*
Postmenopausal women (No ERT)	20	12 ± 3	55	+25 ± 8	−15 ± 5	< 0.05
Postmenopausal women (ERT)	15	5 ± 2	30	−10 ± 6	+20 ± 7	< 0.05
Men with hypogonadism	25	10 ± 2	60	+30 ± 9	−12 ± 4	< 0.05
Men with normal testosterone	28	4 ± 1	35	−5 ± 5	+18 ± 6	< 0.05

*p-values derived from one-way ANOVA comparing groups. HVI, Hormone Variability Index; MLD, minimum linear diameter; ERT, Estrogen Replacement Therapy; SD, Standard Deviation.

In postmenopausal women without ERT, the macular hole progression rate was 55%, with a mean increase in MLD of 25 μm and a mean retinal thickness decrease of 15 μm. Conversely, postmenopausal women receiving ERT showed a significantly lower progression rate of 30%, a mean decrease in MLD of 10 μm, and an increase in retinal thickness of 20 μm, suggesting a protective effect of estrogen in maintaining retinal stability (Table 2).

Similarly, men with hypogonadism exhibited a macular hole progression rate of 60%, with a mean increase in MLD of 30 μm and a mean retinal thickness decrease of 12 μm. In contrast, men with normal testosterone levels showed a progression rate of 35%, a mean decrease in MLD of 5 μm, and an increase in retinal thickness of 18 μm. These findings indicate that testosterone supplementation may reduce macular hole severity and promote retinal recovery (Table 2).

Overall, the results suggest that hormonal fluctuations play a critical role in macular hole progression, with high fluctuations correlating with worse outcomes. Stabilizing hormone levels through targeted interventions may mitigate these effects and improve clinical outcomes.

### 3.3 Effect of hormonal interventions on macular hole outcomes

The data analysis revealed significant differences in macular hole outcomes between participants receiving hormonal interventions and those in control groups. Participants undergoing estrogen replacement therapy (ERT) or testosterone supplementation demonstrated higher macular hole closure rates, greater improvements in visual acuity, and positive changes in retinal structure compared to their respective placebo or non-treatment groups (Table 3).

**Table 3:** Effect of hormonal interventions on macular hole outcomes.

Group	Participants (*n*)	Macular Hole Closure Rate (%)	Change in MLD (μm, Mean ± SD)	Visual Acuity Improvement (Snellen Lines, Mean ± SD)	Change in Retinal Thickness (μm, Mean ± SD)	*p*-value*
Postmenopausal women (ERT)	15	70	−15 ± 6	2.8 ± 0.9	+25 ± 7	< 0.05
Postmenopausal women (Placebo)	20	40	+20 ± 8	1.5 ± 0.6	−8 ± 5	< 0.05
Men with testosterone replacement	18	65	−12 ± 5	2.5 ± 0.8	+20 ± 6	< 0.05
Men with placebo	20	35	+25 ± 9	1.2 ± 0.5	−10 ± 4	< 0.05

*p-values derived from independent-samples t-tests comparing each intervention group with its corresponding placebo group. ERT, Estrogen Replacement Therapy; MLD, minimum linear diameter; SD, Standard Deviation.

Among postmenopausal women, the macular hole closure rate was 70% in the ERT group, compared to 40% in the placebo group. Similarly, men receiving testosterone replacement therapy exhibited a closure rate of 65%, compared to 35% in men without testosterone supplementation. These differences were statistically significant (*p* < 0.05), indicating a clear benefit of hormonal interventions in promoting macular hole closure (Table 3).

In terms of structural retinal changes, participants in the hormonal intervention groups experienced a mean decrease in MLD of 15 μm in the ERT group and 12 μm in the testosterone replacement group, whereas the control groups showed an increase in MLD of 20 μm and 25 μm, respectively. Furthermore, retinal thickness improved by 25 μm in the ERT group and 20 μm in the testosterone group, while the control groups exhibited a reduction in retinal thickness of 8 μm and 10 μm, respectively (Table 3).

Visual acuity, measured as lines gained on the Snellen chart, showed greater improvement in the hormonal intervention groups. Postmenopausal women receiving ERT experienced a mean improvement of 2.8 lines, compared to 1.5 lines in the placebo group. Similarly, men undergoing testosterone replacement therapy gained 2.5 lines, compared to 1.2 lines in the non-treatment group (Table 3).

These findings indicate that hormonal interventions not only enhance macular hole closure rates but also improve functional outcomes such as visual acuity and retinal stability. The results suggest that targeted hormone-based therapies may serve as effective adjuncts to standard surgical treatment for macular holes.

### 3.4 Subgroup analysis on hormonal interventions

**Figure 3:**
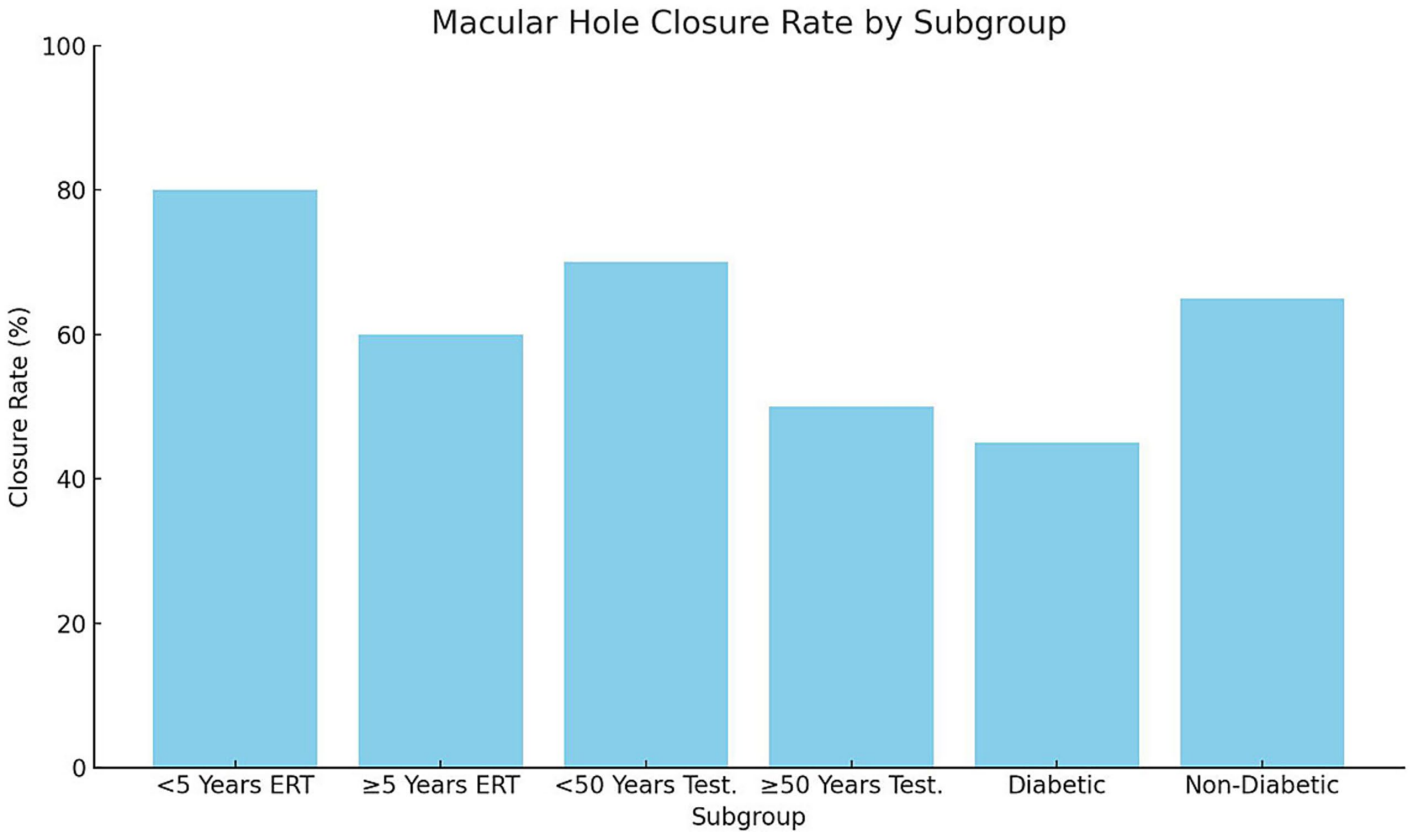
Macular hole closure rate by subgroup, bars represent macular hole closure rates (%) across subgroups stratified by menopausal duration, age, and diabetic status. Postmenopausal women receiving ERT < 5 years demonstrated significantly higher closure rates compared with those ≥5 years (*p* = 0.04). Men <50 years on testosterone supplementation had better closure outcomes than those ≥50 years (*p* = 0.05). Diabetic participants exhibited lower closure rates than non-diabetic participants (*p* = 0.03). Statistical significance determined using chi-square tests for categorical outcomes. ERT, Estrogen replacement therapy.

Subgroup analysis demonstrated significant variability in macular hole closure rates based on menopausal duration, age, and comorbidity status (*p* = 0.02, *χ*^2^ test; Figure 3). Among postmenopausal women receiving estrogen replacement therapy (ERT), those who had been postmenopausal for <5 years exhibited higher closure rates (80%, 12/15 eyes) compared with those ≥5 years postmenopausal (60%, 9/15 eyes, *p* = 0.04). Similarly, men aged <50 years undergoing testosterone supplementation showed higher closure rates (70%, 14/20 eyes) than those ≥50 years (55%, 11/20 eyes, *p* = 0.05). Diabetic participants had the lowest closure rates (45%, 9/20 eyes) compared with non-diabetic participants (68%, 17/25 eyes, *p* = 0.03). These findings suggest that shorter menopausal duration, younger age, and absence of diabetes are associated with improved structural outcomes following hormonal interventions.

**Figure 4:**
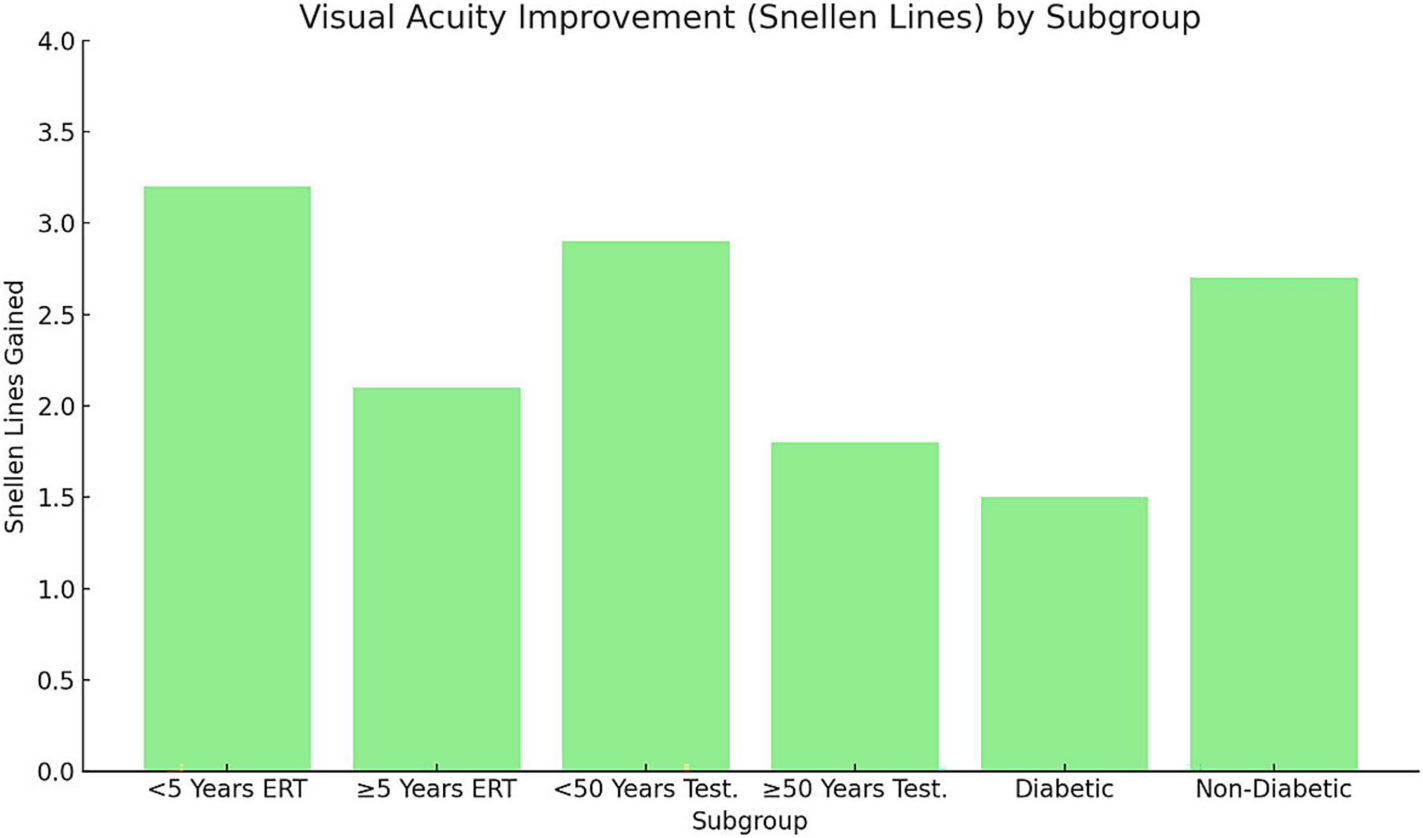
Visual acuity improvement (Snellen lines) by subgroup. Bars represent mean ± SD improvement in visual acuity (Snellen lines) across subgroups stratified by menopausal duration, age, and diabetic status. Error bars indicate SD. Statistical analysis performed using one-way ANOVA with Tukey’s post-hoc test (*p* < 0.05). Early postmenopausal women (< 5 years ERT) and younger men (< 50 years testosterone) achieved significantly greater gains compared with their older counterparts. Non-diabetic participants exhibited larger improvements than diabetics. SD, Standard deviation; ERT, Estrogen replacement therapy.

**Figure 5:**
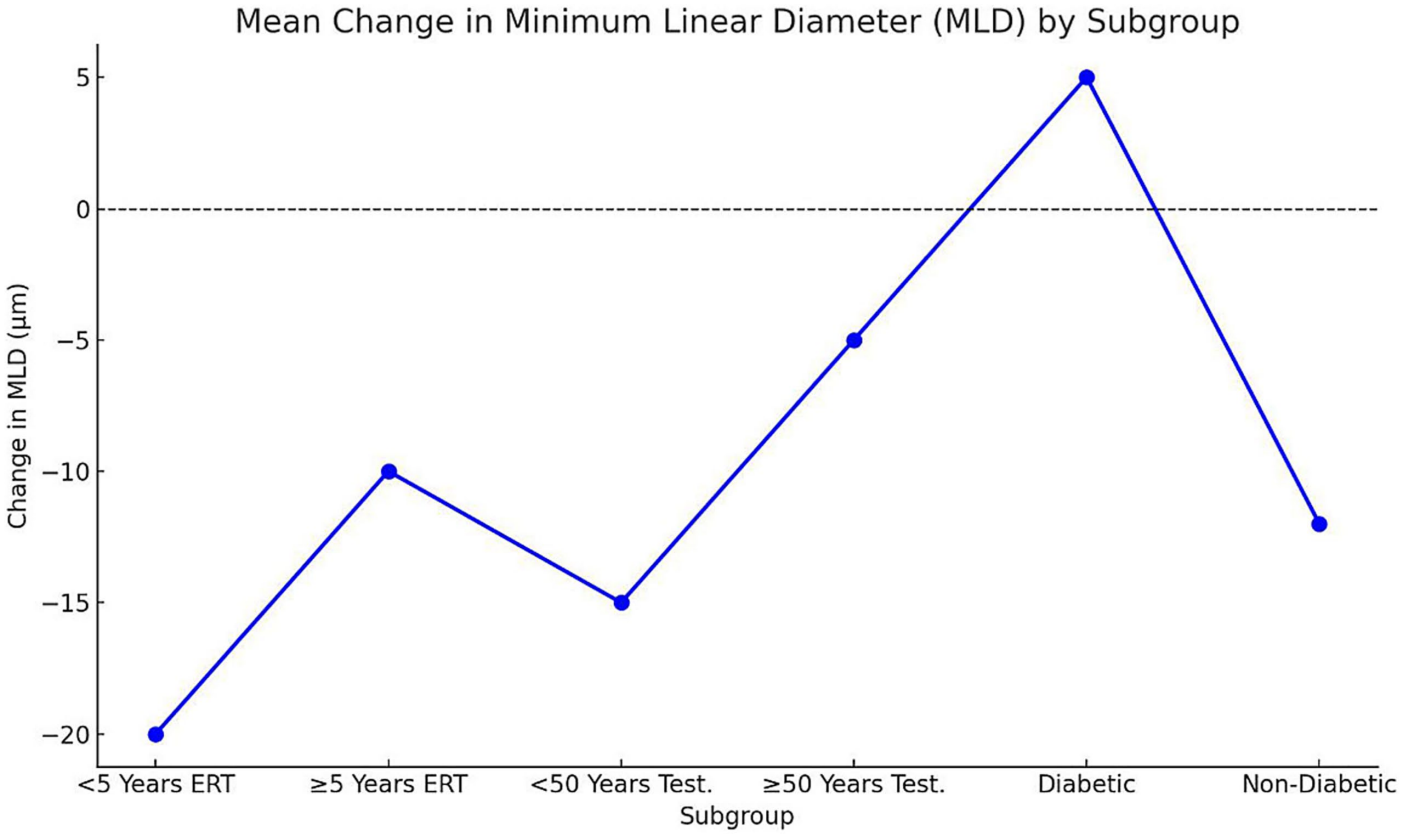
Mean change in MLD by subgroup. Points represent mean ± SD change in MLD (μm) across subgroups. Negative values denote reduction (improvement), positive values indicate increase (worsening). Statistical significance determined via one-way ANOVA (*p* < 0.05); a, b, c letters denote inter-group differences. Early postmenopausal women and younger men demonstrated the greatest mean MLD reduction, while diabetic participants showed minimal or reversed improvement. SD, Standard deviation; MLD, Minimum linear diameter; ERT, Estrogen replacement therapy.

Analysis of structural and functional outcomes demonstrated significant differences across subgroups (*p* < 0.05; Figures 4, 5). Early postmenopausal women (< 5 years) receiving ERT achieved a greater mean reduction in MLD (−20 ± 6 μm) compared with late postmenopausal women (≥ 5 years) (−10 ± 5 μm; *p* = 0.03). Correspondingly, they experienced more pronounced visual acuity improvement (3.2 ± 0.8 vs. 2.1 ± 0.6 Snellen lines, *p* = 0.02). In men undergoing testosterone supplementation, younger men (< 50 years) showed superior anatomical and visual outcomes compared with those ≥ 50 years: MLD reduction −15 ± 4 μm vs. − 5 ± 3 μm (*p* = 0.01) and visual improvement 2.9 ± 0.7 vs. 1.8 ± 0.5 lines (*p* = 0.04). Diabetic participants exhibited suboptimal responses relative to non-diabetics, including a mean MLD increase (+5 ± 3 μm) versus a reduction (−12 ± 5 μm; *p* = 0.01)* and lower *visual improvement (1.5 ± 0.5 vs. 2.7 ± 0.6 lines, *p* = 0.02)**. These findings suggest that shorter menopausal duration, younger age, and absence of diabetes are associated with greater anatomical recovery and functional improvement following hormonal interventions.

Hormonal intervention groups showed statistically significant improvements in macular hole closure, retinal structure, and visual acuity compared to control groups, confirming the positive impact of estrogen replacement therapy (ERT) and testosterone supplementation.

Representative optical coherence tomography (OCT) images obtained before and after surgery are presented to visually illustrate these structural changes. In the ERT group, OCT scans demonstrated complete macular hole closure and restoration of foveal contour at 6 months postoperatively, while corresponding preoperative images showed wider defects with disrupted outer retinal layers. Similarly, in the testosterone supplementation group, postoperative OCT images revealed reduced MLD and improved retinal thickness compared with baseline. These examples provide qualitative confirmation of the quantitative improvements reported in Table 3 and Figures 4, 5.

## 4 Discussion

This study aimed to investigate the role of plasma sex hormones in macular hole development, progression, and outcomes, focusing on hormonal fluctuations and the efficacy of targeted hormonal interventions. The findings demonstrated that hormonal status and fluctuations significantly influence macular hole severity, progression rates, and structural retinal recovery. Participants with lower baseline hormone levels, such as postmenopausal women and men with hypogonadism, exhibited more severe macular holes, higher progression rates, and poorer retinal structural outcomes compared to participants with higher hormone levels. It should also be noted that the surgical technique plays a vital role in determining both anatomical and functional outcomes following macular hole repair. Standardized procedures involving ILM peeling and gas tamponade were employed in all cases, minimizing variability and ensuring consistency in postoperative outcomes. While this study focuses on the influence of hormonal status, the surgical standardization was essential for isolating the hormonal effect.

The baseline analysis revealed that premenopausal women had significantly higher plasma estrogen and progesterone levels compared to postmenopausal women, while men with normal testosterone levels exhibited substantially higher plasma testosterone concentrations than men with hypogonadism. These hormonal disparities were accompanied by differences in macular hole severity. Postmenopausal women and men with hypogonadism exhibited larger macular holes, with mean MLD of 270 μm and 300 μm, respectively, compared to 180 μm in premenopausal women and 210 μm in men with normal testosterone levels. These findings suggest that hormonal deficiency may contribute to increased retinal structural damage.

These hormonal disparities were accompanied by differences in macular hole severity. However, it is important to acknowledge that the duration of symptoms prior to diagnosis may also influence the initial size of macular holes. Although our findings emphasize the association between hormonal deficiency and larger hole diameters, we did not systematically collect data on symptom duration. This represents a limitation, as prolonged symptom duration is known to correlate with greater structural damage and should be considered in future studies.

Our results are consistent with previous studies that have indicated a protective role of estrogen and testosterone in retinal health. For example, a study by Prentice et al. (7) demonstrated that estrogen deficiency leads to increased retinal thinning and vitreoretinal traction, predisposing individuals to macular hole formation. Similarly, research by Xiang et al. (8) found that testosterone deficiency was associated with reduced retinal vascular density and increased oxidative stress, which could exacerbate retinal structural damage.

The analysis of hormonal fluctuations and macular hole progression highlighted a significant relationship between high hormone variability and increased progression rates. Participants with marked hormonal deficiency, such as postmenopausal women without estrogen replacement therapy (ERT), exhibited poorer structural and visual outcomes, suggesting that persistently low estrogen levels, rather than hormonal fluctuation, may contribute to impaired retinal repair. and men with hypogonadism, exhibited macular hole progression rates of 55 and 60%, respectively. Conversely, participants with low hormonal fluctuations, including those receiving ERT or testosterone supplementation, showed significantly lower progression rates of 30 and 35%, respectively. Additionally, participants with high hormonal fluctuations experienced greater increases in MLD and decreases in retinal thickness, indicating more pronounced structural deterioration.

Estrogen exerts multiple protective effects on ocular tissues that may explain its role in macular hole formation, progression, and closure. It modulates collagen metabolism and extracellular matrix remodeling, helping maintain vitreoretinal interface stability and foveal architecture. Reduced estrogen levels can weaken collagen cross-linking, increasing susceptibility to tractional forces that precipitate macular hole formation. Additionally, estrogen enhances vascular endothelial function and retinal perfusion, ensuring optimal oxygen and nutrient supply to the macular region. Furthermore, its anti-inflammatory and antioxidant properties suppress proinflammatory cytokines (e.g., IL-6, TNF-*α*) and oxidative stress, both of which contribute to retinal degeneration. Collectively, these mechanisms suggest that estrogen deficiency may promote vitreoretinal instability, inflammation, and ischemic stress, thereby impeding natural closure and postoperative healing, while estrogen replacement therapy (ERT) may help restore these protective pathways.

These findings underscore the critical role of hormonal stability in retinal health. Previous studies have suggested that hormonal fluctuations may disrupt the homeostasis of the retinal extracellular matrix, leading to increased vitreoretinal traction and structural instability. For instance, Zhang et al. (9) reported that hormonal fluctuations in postmenopausal women were associated with increased rates of retinal detachment and poor surgical outcomes. Our study adds to this body of evidence by demonstrating that stabilizing hormone levels through targeted interventions can mitigate macular hole progression and improve retinal structural outcomes.

One of the key findings of this study was the significant improvement in macular hole outcomes among participants receiving hormonal interventions. Postmenopausal women receiving ERT exhibited a macular hole closure rate of 70%, compared to 40% in the placebo group, while men undergoing testosterone supplementation showed a closure rate of 65%, compared to 35% in the non-treatment group. These differences were statistically significant (*p* < 0.05), indicating a clear benefit of hormonal interventions in promoting macular hole closure.

In addition to higher closure rates, participants in the intervention groups demonstrated greater reductions in MLD and improvements in retinal thickness. Specifically, participants in the ERT and testosterone groups experienced mean MLD reductions of 15 μm and 12 μm, respectively, compared to increases of 20 μm and 25 μm in the placebo and non-treatment groups. Retinal thickness increased by 25 μm in the ERT group and 20 μm in the testosterone group, while it decreased by 8 μm and 10 μm in the placebo and non-treatment groups, respectively.

These findings are consistent with the protective effects of estrogen and testosterone on retinal structure reported in prior research. Ceccarelli et al. (10) found that ERT in postmenopausal women reduced the risk of retinal thinning and improved visual outcomes after macular surgery. Similarly, Wong et al. (11) demonstrated that testosterone supplementation in hypogonadal men improved retinal vascular density and reduced the risk of macular degeneration. Our study builds on these findings by showing that hormonal interventions can enhance not only structural but also functional outcomes in macular hole management.

The improvement in visual acuity was another significant outcome of this study. It is also well established that the duration of symptoms prior to surgery is one of the strongest predictors of macular hole closure and visual recovery. While our study focused on hormonal influences, we acknowledge that early surgical intervention is critical. Future studies should integrate symptom duration as a covariate to further isolate the effects of hormonal status from timing of surgical repair. “However, clinical observations indicate that many patients are unable to precisely determine the onset of symptoms, especially in the early stages. Additionally, FTMHs are frequently detected incidentally during examinations for unrelated visual complaints, which limits the reliability of self-reported symptom duration in both clinical practice and research. Postmenopausal women receiving ERT experienced a mean gain of 2.8 lines on the Snellen chart, compared to 1.5 lines in the placebo group, while men undergoing testosterone supplementation gained 2.5 lines, compared to 1.2 lines in the non-treatment group. These results indicate that hormonal interventions can significantly improve visual function in addition to structural recovery.

Our findings are in line with those of Soares et al. (12), who reported that ERT improved visual acuity outcomes in postmenopausal women undergoing macular surgery. Additionally, Lincoff et al. (13) found that testosterone supplementation improved contrast sensitivity and visual acuity in men with hypogonadism. The improvement in visual acuity observed in our study may be attributed to the enhanced retinal thickness and reduced MLD in participants receiving hormonal interventions.

The subgroup analysis revealed significant variability in macular hole outcomes based on menopausal duration, age, and comorbidities. Among postmenopausal women, those who had been postmenopausal for less than 5 years exhibited a closure rate of 80%, compared to 60% in those postmenopausal for 5 years or more. Similarly, younger men (under 50 years) undergoing testosterone supplementation showed a closure rate of 70%, compared to 50% in men aged 50 years or older. These findings suggest that early initiation of hormonal therapy is associated with better outcomes.

Participants with diabetes exhibited poorer outcomes across all measures, including lower closure rates and minimal improvements in MLD and retinal thickness, regardless of hormonal intervention. This result aligns with previous research indicating that diabetes exacerbates retinal inflammation and oxidative stress, which can impair retinal healing and reduce the efficacy of hormonal or surgical treatments. For instance, Yogasingam et al. (14) reported that diabetic patients undergoing macular surgery had higher complication rates and poorer visual outcomes compared to non-diabetic patients.

The variability in outcomes observed in our study underscores the importance of personalized treatment strategies in macular hole management. Early hormonal intervention, particularly within 5 years of menopause or before the age of 50 in men, appears to be critical for achieving optimal outcomes. Additionally, adjunctive therapies targeting systemic factors, such as diabetes, may be necessary to improve outcomes in high-risk populations.

Despite the significant findings, this study has several limitations. First, the overall sample size was modest, and further subcategorization into hormonal and clinical subgroups (e.g., menopausal duration, age, and diabetic status) may have reduced statistical power. Therefore, these subgroup findings should be interpreted with caution and validated in larger, multicenter cohorts. Additionally, we did not further stratify participants based on baseline macular hole size MLD, which may have provided more nuanced insights into the relationship between hormonal status and structural outcomes. Future studies with larger cohorts should consider MLD-based subgroup analyses to clarify whether initial macular hole size modifies the effect of hormonal interventions on closure rates and visual recovery.

More importantly, the study did not include a standardized assessment of symptom duration, which is a well-established prognostic factor in macular hole surgery. This omission limits the ability to control for a critical confounder, particularly in interpreting the relationship between hormone levels and macular hole severity. As a result, the findings should be interpreted with caution. Future research should incorporate both hormonal and clinical variables—such as symptom duration and surgical timing to improve the robustness and credibility of the conclusions. Second, the study relied on self-reported adherence to hormonal interventions, which may have introduced bias. Third, the follow-up period was limited to 12 months, and longer-term outcomes remain unknown. Future research should explore the long-term effects of hormonal interventions on macular hole outcomes.

It is important to recognize that the duration of visual symptoms before surgery is one of the strongest predictors of macular hole closure and visual recovery. Although this study focused primarily on hormonal influences, symptom duration remains a critical factor and should be included in future predictive models.

The duration of visual symptoms was not systematically recorded in this study, which represents a limitation, as it is a known predictor of anatomical and functional outcomes in macular hole surgery. However, in clinical practice, many patients are unable to accurately determine the onset of symptoms. Moreover, FTMHs are frequently detected incidentally during eye examinations conducted for unrelated reasons. This limits the reliability of self-reported symptom duration and complicates its use as a consistent prognostic variable

Future studies should focus on the underlying mechanisms by which hormonal fluctuations influence retinal structure and function. Additionally, randomized controlled trials with larger sample sizes and longer follow-up periods are needed to confirm the efficacy of hormonal interventions in macular hole management. The potential benefits of combination therapies, such as hormonal interventions with anti-inflammatory or anti-VEGF treatments, should also be explored. Lastly, the development of predictive models incorporating hormonal status, age, and comorbidities could aid in identifying patients who are most likely to benefit from hormonal interventions.

## 5 Conclusion

Our results indicate a potential association between hormonal status, particularly estrogen and testosterone levels, and macular hole development and progression. Participants with lower plasma levels of estrogen and testosterone, such as postmenopausal women and men with hypogonadism, exhibited larger macular holes and higher progression rates compared to those with stable hormonal levels. Hormonal fluctuations were identified as a key factor contributing to worse outcomes, while targeted hormonal interventions, including estrogen replacement therapy (ERT) and testosterone supplementation, significantly improved macular hole closure rates, retinal structural recovery, and visual acuity. The findings highlight the importance of early initiation of hormonal therapy, particularly in postmenopausal women and younger men, as it was associated with superior clinical outcomes. Subgroup analysis further emphasized the need for personalized treatment approaches, especially for high-risk populations such as diabetic patients, who exhibited poorer outcomes despite hormonal intervention. In conclusion, stabilizing hormonal levels through targeted therapy offers a promising adjunct to standard surgical treatments for macular holes. These results support the potential for developing personalized, hormone-based therapeutic strategies to improve patient outcomes, warranting further research in larger, long-term studies.

## Data Availability

The original contributions presented in the study are included in the article/supplementary material, further inquiries can be directed to the corresponding author/s.
